# Racial and socioeconomic disparities in surgical care for post‐prostate cancer treatment complications: A nationwide Medicare‐based analysis

**DOI:** 10.1002/bco2.342

**Published:** 2024-02-29

**Authors:** Oluwafolajimi Adesanya, Sirikan Rojanasarot, Alysha M. McGovern, Arthur L. Burnett

**Affiliations:** ^1^ Department of Urology, James Buchanan Brady Urological Institute Johns Hopkins University School of Medicine Baltimore Maryland USA; ^2^ Boston Scientific Marlborough Massachusetts USA

**Keywords:** erectile dysfunction, healthcare disparities, prostate cancer, prostatectomy, urinary incontinence

## Abstract

**Objectives:**

To investigate the racial and socioeconomic (income) differences in receipt of and time to surgical care for urinary incontinence (UI) and erectile dysfunction (ED) occurring post‐radical prostatectomy (RP) and/or radiation therapy (RT).

**Materials and Methods:**

Utilizing the Medicare Standard Analytical Files (SAF), a retrospective cohort study was performed on data of patients diagnosed with prostate cancer (PCa) from 2015 to 2021. Patients who underwent RP and/or RT and who subsequently developed UI and/or ED were grouped into four cohorts: RP‐ED, RP‐UI, RT‐ED and RT‐UI. County‐level median household income was cross‐referenced with SAF county codes, classified into income quartiles, and used as a proxy for patient income status. The rate of surgical care was compared between groups using two‐sample t‐test and log‐rank test. Cox proportional hazards modelling was used to determine covariate‐adjusted impact of race on time to surgical care.

**Results:**

The rate of surgical care was 6.8, 3.61 3.07, and 1.54 per 100 person‐years for the RP‐UI, RT‐UI, RP‐ED, and RT‐ED cohorts, respectively. Cox proportional *‘time‐to‐surgical care’* regression analysis revealed that Black men were statistically more likely to receive ED surgical care (RP‐ED AHR:1.79, 95% CI:1.49–2.17; RT‐ED AHR:1.50, 95% CI:1.11–2.01), but less likely to receive UI surgical care (RP‐UI AHR:0.80, 95% CI:0.67–0.96) than White men, in all cohorts except RT‐UI. Surgical care was highest among Q1 (lowest income quartile) patients in all cohorts except RT‐UI.

**Conclusions:**

Surgical care for post‐PCa treatment complications is low, and significantly impacted by racial and socioeconomic (income) differences. Prospective studies investigating the basis of these results would be insightful.

## 1 INTRODUCTION

Prostate cancer (PCa) is the most prevalent malignancy diagnosed in American men.^1^ The 5‐year survival rate has consistently increased from 43% in the mid‐1950s to approximately 100% in 2017 due to advances in therapies and the increased use of prostate specific antigen (PSA) screening.^2^ Radical prostatectomy (RP) and radiation therapy (RT) constitute definitive treatments for localized PCa, with various options, including open retropubic, robotic/laparoscopic or perineal approach for RP, and external beam radiotherapy as well as brachytherapy for RT. Irrespective of the approach used, these procedures may result in complications,^3^ the most prevalent of which are urinary incontinence (UI) and erectile dysfunction (ED).

Orom and colleagues reported significant racial disparities in the occurrence of post‐prostatectomy and post‐radiotherapy UI and ED, with Black men having worse urinary function, while experiencing faster recovery of erectile function post‐radical prostatectomy compared to White men.^4^ Similarly, among those who had RP, men belonging to a lower socioeconomic class had higher rates of sexual and urinary dysfunction compared with those from higher socioeconomic groups.^4^ Among those who had RT, men belonging to lower socioeconomic groups were found to have better sexual function post‐treatment.^4^

There are several options for treating post‐PCa ED including oral phosphodiesterase‐5 inhibitors (PDE‐5i), vasoactive intracorporeal injections or intraurethral suppositories, vacuum erection devices and implantable penile prostheses.^5^ Due to the rising cost of care, some treatment options are being excluded from insurance coverage, requiring higher co‐payments from service beneficiaries. As a result, some experts^6^ have argued that ED treatment is getting costlier, potentially creating racial and socioeconomic disparities in receipt and time to initiation of surgical care for ED in post‐PCa patient populations, with crucial implications for post‐operative quality of life, physical wellbeing, self‐esteem and productivity.

Similarly, the cost of the surgical treatment options for UI has a potential to perpetuate existing racial and socioeconomic disparities in receipt and time to initiation of therapy.^7^ However, while a few previous studies have reported the impact of race,^8^, ^9^ no study has investigated both disparity trends in men with post‐PCa treatment UI. We hypothesized that Black men and men of lower income groups would be less likely to receive ED and UI surgical care compared to White men and those of higher income groups. Thus, we evaluated the receipt of and modelled time to initiation of surgical care for UI and ED post‐PCa treatment, by race and income (as proxy for socioeconomic status).

## 2 PATIENTS AND METHODS

### 2.1 Study design and population

A retrospective cohort study was conducted using the 100% Medicare Standard Analytical Files (SAF). Men diagnosed with PCa, who underwent RP and/or RT, and subsequently experienced ED and/or UI post‐index treatment formed the primary cohort for this study. This population was identified within the SAF database between 2015 and 2021. PCa patients, as well as those who had developed ED and/or UI, were identified using relevant International Classification of Diseases, Ninth Revision (ICD‐9‐CM) and International Classification of Diseases, Tenth Revision (ICD‐10‐CM) codes (Table 1).

**TABLE 1:** Diagnosis and procedure codes.

	HCPCS	CPT code	ICD‐9‐CM	ICD‐10‐CM
**Procedures**				
Radical prostatectomy (RP)		55 810, 55 812, 55 815, 55 840, 55 842, 55 845, 55 866	60.5	
Radiation therapy (RT)	C9728, G6001–G6014	55 876, 55 920, 77 301, 77 385, 77 401, 77 402, 77 412, 77 417, 77 423–77 427, 77 431, 77 432, 77 435, 77 469, 77 470, 77 499, 77 520, 77 522, 77 523, 77 525, 77 761–77 763, 77 767, 77 768, 77 770–77 772, 77 778, 77 789, 77 790, 77 799	92.2 (including 92.21–92.29), V58.0	DV000ZZ, DV001ZZ, DV002ZZ, DV003Z0, DV003ZZ, DV004ZZ, DV005ZZ, DV006ZZ, DV1097Z, DV1098Z, DV1099Z, DV109BZ, DV109CZ, DV109YZ, DV10B6Z, DV10B7Z, DV10B8Z, DV10B9Z, DV10BB1, DV20JZZ, DV10BBZ, DV10BCZ, DV10BYZ, DV20DZZ, DV20HZZ, DVYO7ZZ, DVYO8ZZ, DVYOCZZ, DVYOFZZ, DVYOKZZ
Artificial urinary sphincter (AUS) and male sling insertion	C1815	53 444–53 449		58.93, 0THC0LZ, 0THD0LZ, 0TWB0LZ, 0TWD0LZ, 0TUC0JZ, 0TUD0JZ, OTWB0JZ, 0TWD0JZ
Penile prosthesis implantation—and associated procedure complications^a^	C1813, C2622	00938, 54 400, 54 401, 54 405, 54 406, 54 408, 54 410, 54 411, 54 415–54 417	64.94–64.97	T83.410 (including T83.410A, T83.410D, T83.410S), T83.420 (including T83.420A, T83.420D, and T83.420S), T83.490 (including T83.490A, T83.49D and T83.49S)—associated procedure complication codes^a^
**Diagnoses**				
Malignant neoplasm of the prostate			185	C61, Z85.46
Erectile dysfunction (ED)—including some non‐specific ED codes^b^			607.84	N52.31, N52.35–N52.37, N52.39 as well as N52.8, N52.9, R37, F52, N52.01–N52.03^b^
Urinary incontinence (UI)—mostly non‐specific codes^b^			788.3, 788.31–788.39, 788.91^b^	N39.3, N39.41–N39.46, N39.49, N39.490, N39.491, N39.498^b^

Abbreviations: CPT, current procedural terminology; ICD‐9/10‐CM, international classification of diseases, 9th/10th clinical modification; HCPCS, healthcare common procedural coding system.

^a^ These are codes for complication associated with penile prosthesis, AUS, male sling insertion, and are only included if they appear for the first time after the prostate cancer diagnosis.

^b^ These are non‐specific codes, that is, not specific for post‐RP or RT periods, and thus are only included if they appear for the first time after the index treatment, that is, RP or RT.

Patients who had undergone relevant ED and/or UI treatment procedures were identified using the corresponding Current Procedural Terminology (CPT), and Healthcare Common Procedure Coding System (HCPCS) procedure codes (Table 1). Patients were grouped based on index treatment and post‐treatment complication into four cohorts: RP‐ED (patients with ED following RP; *n* = 11 567), RP‐UI (patients with UI following RP; *n* = 12 100), RT‐ED (patients with ED following RT; *n* = 8358) and RT‐UI (patients with UI following RT; *n* = 5329).

**FIGURE 1:**
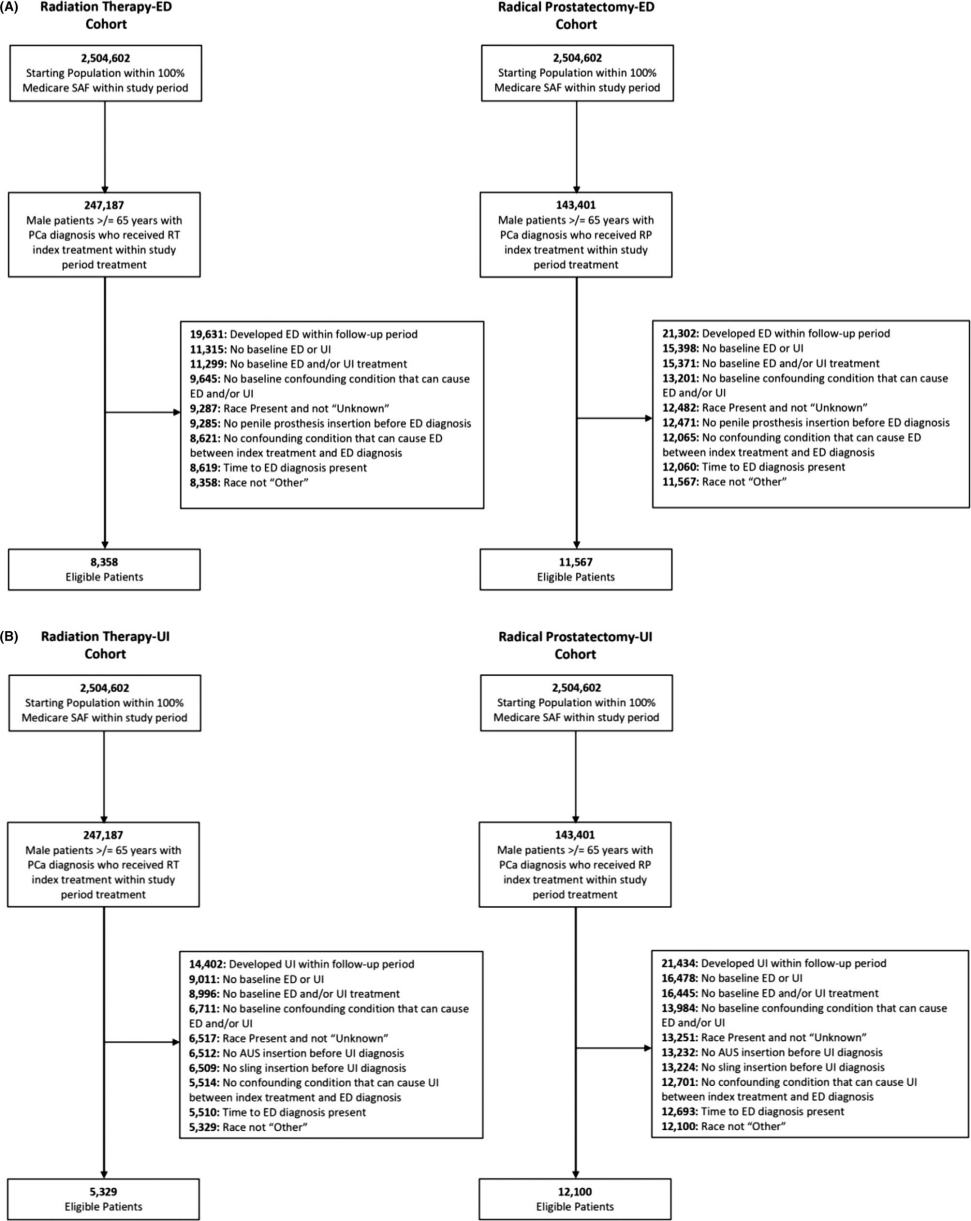
Cohort attrition flowcharts. (A) Radiation Therapy‐Erectile Dysfunction (RT‐ED) and Radical Prostatectomy‐Erectile dysfunction (RP‐ED) cohort attrition flowcharts, and (B) Radiation Therapy‐Urinary Incontinence (RT‐UI) and Radical Prostatectomy‐Urinary Incontinence (RP‐UI) cohort attrition flowchart.

Patients with incomplete data points and those with ED and/or UI diagnosis codes prior to their PCa diagnosis were excluded from the study. Patients who died before the end of the study period, those who disenrolled from Medicare, or those who were diagnosed with a confounding chronic condition (including dementia, multiple sclerosis, Parkinson's disease, spinal cord injury, stroke and seizure disorder) which could cause ED and/or UI independent of PCa, either prior to or during the study period, were censored during the analysis (Figure 1A,B). Since this study does not involve human participants, neither institutional review board approval nor participant consent was obtained.

### 2.2 Outcomes

The primary study outcomes were the receipt of ED and/or UI surgical care within the study period, as well as the time to initiation of care from complication diagnosis. The surgical treatment options evaluated include penile prostheses (PP) for ED and artificial urinary sphincter (AUS) or male sling insertion for UI. Time to surgical care was defined as duration from complication diagnosis (defined as date of first UI/ED ICD‐9‐CM/ICD‐10‐CM record) to receipt of the first surgical intervention (defined as date of PP/AUS/male sling CPT/HCPCS procedural code record).

### 2.3 Exposure variables

The two exposure variables evaluated were race and income. Race was extracted from the 100% Medicare SAF and categorized as White or Black. County‐level 2021 median household income (MHI) data were obtained from the United States Census Bureau Small Area Income and Poverty Estimates (SAIPE) dataset, crossmatched with the SAF database individual patient county codes, classified into quartiles: Q1 (<$47 903), Q2 ($47 903–$55 310), Q3 ($55 311–$64 309) and Q4 (>$64 309) and used as a proxy for patient income groups.

### 2.4 Covariates

Patient demographic information, including age, geographic location (classified as Midwest, Northeast, South or West) and co‐morbidities (evaluated using the Charlson and Elixhauser comorbidity indices), were collected and analysed as covariates. Charlson and Elixhauser comorbidity indices at baseline were determined from the Medicare inpatient claims data available within the database in the 12 months prior to the PCa diagnosis.

### 2.5 Statistical analysis

Continuous variables are presented as mean ± standard deviation (SD), while categorical variables are presented as percentages. Intra‐cohort baseline characteristics of the study population across racial and income groups were examined using the chi‐square test for categorical variables and the t‐test or ANOVA for continuous variables. Intra‐cohort rates of surgical care (per 100 person‐years) with corresponding 95% confidence intervals (CI) were calculated and compared across racial and income groups using the t‐test and ANOVA, respectively. Statistical significance was set at *p* < 0.05. Covariate‐adjusted effects of race on the time to surgical care were determined through Cox proportional hazard regression modelling, with age, geographical location and Charlson comorbidity score as the key covariates. The precision of adjusted hazard ratios (AHRs) was assessed using corresponding 95% CIs. All analyses were performed using the Instant Health Data (IHD) software (Panalgo, Boston MA, USA) and R, version 3.2.1 (R Foundation for Statistical Computing, Vienna, Austria).

## 3 RESULTS

### 3.1 Baseline characteristics

A total of 37 354 eligible patients were identified, consisting of 33 780 White and 3574 Black men, who were grouped into the four study cohorts. Table 2 presents the intra‐cohort patient demographic characteristics and comorbidity indices. There were significant intra‐cohort differences in all baseline characteristics across races. Black men were younger (RP‐ED: 68.7 vs. 67.9 years, RP‐UI: 69.3 vs. 68.4 years, RT‐ED: 71.1 vs. 69.8 years, RT‐UI: 73.1 vs. 71.8 years; all *p* < 0.05) and had higher mean Elixhauser (RP‐ED: 3.9 vs. 3.4, RP‐UI: 3.7 vs. 4.1, RT‐ED: 3.6 vs. 4.3, RT‐UI: 4.4 vs. 4.9; all *p* < 0.05) as well as higher mean Charlson (RP‐ED: 2.8 vs. 3.0, RP‐UI: 2.9 vs. 3.1, RT‐ED: 3.1 vs. 3.4, RT‐UI: 3.1 vs. 4.1; all *p* < 0.05) comorbidity scores at complication diagnosis in all cohorts (Table 3). Men in the lowest income quartile were younger and had significantly higher mean Elixhauser scores in all cohorts at complication diagnosis. This was also observed for the intra‐cohort comparison of mean Charlson comorbidity scores across income groups; however, the observed pattern was not statistically significant (Table 4).

**TABLE 2:** Baseline characteristics.

Variable	RP‐ED (*n* = 11 567)	RP‐UI (*n* = 12 100)	RT‐ED (*n* = 8358)	RT‐UI (*n* = 5329)
**Age**				
*N*	11 567	12 100	8358	5329
*Mean (SD)*	68.7 (3.2)	69.3 (3.5)	70.9 (4.6)	73.0 (5.9)
*Median (IQR)*	68 (66–71)	69 (67–71)	70 (67–74)	72 (68–76)
*Min‐Max*	65–91	65–91	65–93	65–99
**Race** *n (%)*				
*White*	10 543 (91.2)	11 168 (92.3)	7225 (86.4)	4844 (90.9)
*Black*	1024 (8.9)	932 (7.7)	1133 (13.6)	485 (9.1)
**County Median Household Income** *$US*				
*N*	11 545	12 076	8334	5321
*Mean (SD)*	68 502 (17695)	67 878 (17348)	68 291 (18741)	68 117 (18487)
*Median (IQR)*	64 931 (55 688–76 409)	64 501 (55 402–75 826)	64 371 (55 011–76 424)	64 526 (55 102–76 287)
**Income Quartile** *n (%)*				
*Q1*	860 (7.5)	975 (8.1)	768 (9.2)	528 (9.9)
*Q2*	1940 (16.8)	2014 (16.7)	1476 (17.7)	877 (16.5)
*Q3*	2814 (24.4)	2974 (24.7)	1892 (22.7)	1211 (22.8)
*Q4*	5925 (51.4)	6102 (50.6)	4195 (50.4)	2704 (50.8)
**Patient Census Region** *n (%)*				
*Midwest*	3066 (26.6)	3246 (26.9)	2025 (24.3)	1423 (26.7)
*Northeast*	1936 (16.8)	1923 (15.9)	1641 (19.7)	1055 (19.8)
*South*	3955 (34.3)	4422 (36.7)	3173 (38.1)	1820 (34.2)
*West*	2585 (22.4)	2475 (20.5)	1498 (18.0)	1023 (19.2)
**Elixhauser Comorbidity Score**				
*N*	11 567	12 100	8358	5329
*Mean (SD)*	3.5 (2.1)	3.7 (2.2)	3.7 (2.5)	4.5 (2.9)
*Median (IQR)*	3 (2–4)	3 (2–5)	3 (2–5)	4 (2–6)
*Min‐Max*	0–17	0–17	0–19	0–20
**Charlson Comorbidity Score**				
*N*	11 567	12 100	8358	5329
*Mean (SD)*	2.8 (1.4)	2.9 (1.5)	3.1 (1.8)	3.7 (2.2)
*Median (IQR)*	2 (2–3)	2 (2–3)	2 (2–4)	3 (2–6)
*Min‐Max*	0–12	0–13	0–17	0–14

Abbreviations: ED, erectile dysfunction; IQR, interquartile range; RP, radical prostatectomy; RT, radiation therapy; UI, urinary incontinence; SD, standard deviation.

**TABLE 3:** Baseline characteristics by race.

Variable	RP‐ED (*n* = 11 567)			RP‐UI (*n* = 12 100)			RT‐ED (*n* = 8358)			RT‐UI (*n* = 5329)		
	White (*n* = 10 543)	Black (*n* = 1024)	*p*	White (*n* = 11 168)	Black (*n* = 932)	*p*	White (*n* = 7225)	Black (*n* = 1133)	*p*	White (*n* = 4844)	Black (*n* = 485)	*p*
**Age**												
*Mean (SD)*	68.7 (3.2)	67.9 (2.8)	**<0.0001** *	69.3 (3.5)	68.4 (3.1)	**<0.0001** *	71.1 (4.6)	69.8 (4.2)	**<0.0001** *	73.1 (5.9)	71.8 (5.5)	**<0.0001** *
*Median*	68.0	67.0	‐	69.0	68.0	‐	70.0	69.0	‐	72.0	71.0	‐
**Elixhauser Comorbidity Score**												
*Mean (SD)*	2.8 (1.4)	3.0 (1.6)	**<0.0001** *	2.9 (1.5)	3.1 (1.7)	**<0.0001** *	3.1 (1.8)	3.4 (1.9)	**<0.0001** *	3.7 (2.2)	4.1 (2.4)	0.0021
*Median*	2.0	2.0	‐	2.0	2.0	‐	2.0	2.0	‐	3.0	3.0	‐
**Charlson Comorbidity Score**												
*Mean (SD)*	2.8 (1.4)	3.0 (1.6)	**<0.0001** *	2.9 (1.5)	3.1 (1.7)	0.0024	3.1 (1.8)	3.4 (1.9)	**<0.0001** *	3.7 (2.2)	4.1 (2.4)	0.0019
*Median*	2.0	2.0	‐	2.0	2.0	‐	2.0	2.0	‐	3.0	3.0	‐
**Patient Census Region** *n [approx.,000] [%]*												
*Midwest*	2.9 (27.1)	0.2 (21.3)		3.1 (27.5)	0.2 (20.3)		1.8 (25.4)	0.2 (17.2)		1.4 (27.6)	0.09 (17.7)	
*Northeast*	1.8 (16.9)	0.2 (15.2)		1.8 (16.1)	0.1 (14.3)		1.5 (20.5)	0.2 (14.6)		1.0 (20.3)	0.07 (15.2)	
*South*	3.4 (32.2)	0.6 (55.9)		3.9 (35.0)	0.5 (57.2)		2.5 (34.4)	0.7 (61.6)		1.5 (32.0)	0.3 (56.9)	
*West*	2.5 (23.8)	0.08 (7.7)		2.4 (21.5)	0.08 (8.1)		1.4 (19.7)	0.08 (6.7)		1.0 (20.1)	0.05 (10.2)	

Abbreviations: ED, erectile dysfunction; RP, radical prostatectomy; RT, radiation therapy; UI, urinary incontinence; SD, standard deviation.

* Statistically significant at *p* ≤ 0.05.

**TABLE 4:** Baseline characteristics by income quartile.

Cohort	Income auartile	N	Age (years)			Elixhauser Comorbidity Score			Charlson Comorbidity Score			Patient Census Region *n (%)*			
			Mean (SD)	Median (IQR)	*p*	Mean (SD)	Median (IQR)	*p*	Mean (SD)	Median (IQR)	*p*	Midwest	Northeast	South	West
**RP‐ED**	Q1	860	68.54 (3.24)	68 (66–70)	0.0006*	3.69 (2.14)	3 (2–5)	<0.0001*	2.80 (1.37)	2 (2–3)	0.3521	102 (11.86)	25 (3.26)	610 (79.43)	42 (5.47)
	Q2	1940	68.49 (3.10)	68 (66–70)		3.67 (2.14)	3 (2–5)		2.84 (1.44)	2 (2–3)		480 (32.52)	163 (11.04)	650 (44.04)	183 (12.40)
	Q3	2814	68.65 (3.15)	68 (66–71)		3.47 (2.05)	3 (2–4)		2.76 (1.40)	2 (2–3)		601 (31.82)	222 (11.73)	811 (42.86)	257 (13.58)
	Q4	5925	68.76 (3.20)	68 (66–71)		3.33 (2.03)	3 (2–4)		2.79 (1.47)	2 (2–3)		852 (20.31)	1231 (29.34)	1098 (26.17)	1014 (24.17)
**RP‐UI**	Q1	975	69.15 (3.56)	68 (66–71)	0.032*	3.95 (2.26)	3 (2–5)	<0.0001*	2.98 (1.49)	2 (2–3)	0.0826	144 (14.77)	30 (3.08)	728 (74.67)	73 (7.49)
	Q2	2014	69.13 (3.49)	68 (66–71)		3.89 (2.26)	3 (2–5)		2.93 (1.50)	2 (2–3)		630 (31.28)	199 (9.88)	939 (46.62)	246 (12.21)
	Q3	2974	69.20 (3.43)	69 (66–71)		3.69 (2.19)	3 (2–5)		2.87 (1.51)	2 (2–3)		1030 (34.63)	299 (10.05)	1129 (37.96)	516 (17.35)
	Q4	6102	69.35 (3.53)	69 (67–71)		3.56 (2.17)	3 (2–5)		2.87 (1.52)	2 (2–3)		1442 (23.63)	1395 (22.86)	1625 (26.63)	1640 (26.88)
**RT‐ED**	Q1	768	70.50 (4.41)	70 (67–73)	0.0003*	3.96 (2.46)	3 (2–5)	0.0006*	3.05 (2.06)	3 (2–5)	0.4847	91 (11.85)	25 (3.26)	610 (79.43)	42 (5.4)
	Q2	1476	70.72 (4.46)	70 (67–74)		3.84 (2.49)	3 (2–5)		3.10 (1.77)	2 (2–4)		480 (32.52)	163 (11.04)	650 (44.04)	183 (12.40)
	Q3	1892	70.78 (4.63)	70 (67–74)		3.76 (2.52)	3 (2–5)		3.10 (1.78)	2 (2–4)		602 (31.82)	222 (11.73)	811 (42.86)	257 (13.58)
	Q4	4195	71.12 (4.66)	70 (67–74)		3.63 (2.44)	3 (2–5)		3.15 (1.79)	2 (2–4)		852 (20.31)	1231 (29.34)	1098 (26.17)	1014 (24.17)
**RT‐UI**	Q1	528	72.38 (5.58)	71 (68–76)	0.0005*	4.82 (2.93)	4 (3–6)	0.0003*	3.67 (2.06)	3 (2–5)	0.7323	75 (14.20)	30 (5.68)	354 (68.94)	59 (11.17)
	Q2	877	72.46 (5.86)	71 (68–76)		4.63 (2.90)	4 (2–6)		3.76 (2.21)	3 (2–6)		284 (32.38)	105 (11.97)	382 (43.56)	106 (12.09)
	Q3	1211	72.89 (5.87)	72 (68–77)		4.48 (2.90)	4 (2–6)		3.71 (2.22)	3 (2–5)		448 (36.99)	158 (13.05)	402 (33.20)	203 (16.76)
	Q4	2704	73.24 (5.87)	72 (69–77)		4.30 (2.85)	4 (2–6)		3.77 (2.22)	3 (2–6)		616 (22.78)	762 (28.18)	671 (24.82)	655 (24.22)

Abbreviations: ED, erectile dysfunction; IQR, interquartile range; RP, radical prostatectomy; RT, radiation therapy; UI, urinary incontinence; SD, standard deviation.

**Q1:** <$47 903; **Q2:** $47 903 to $55 310; **Q3:** $55 311 to $64 309; **Q4:** >$64 309.

* Statistically significant at *p* ≤ 0.05.

### 3.2 Receipt of surgical care for adverse event

Rate of surgical care was 3.1, 7.0, 1.5 and 3.6 per 100 person‐years respectively, for the RP‐ED, RP‐UI, RT‐ED and RT‐UI cohorts. On comparison (Table 5), there was a higher rate of ED surgical care among Black men (RP‐ED: 2.8 [95% CI: 2.6–3.0] vs. 5.8 [95% CI: 4.9–6.9], RT‐ED: 1.4 [95% CI: 1.2–1.6] vs. 2.3 [95% CI: 1.8–3.0]), while White men had higher rates of UI surgical care (RP‐UI: 7.1 [95% CI: 6.8–7.4] vs. 5.7 [95% CI: 4.8–6.7], RT‐UI: 3.7 [95% CI: 3.3–4.1] vs. 3.2 [95% CI: 2.2–4.6]). These intra‐cohort differences across racial groups were significant within all cohorts, except in the RT‐UI cohort (*p* = 0.46). The rate of surgical care was highest among men in the Q1 (lowest) income group in all cohorts except RT‐UI, where it was highest among men in the Q2 (second lowest) income group (Table 6). As determined by a log‐rank test, these intra‐cohort differences across income groups were significant in the RP‐ED cohort alone (*p* < 0.05).

**TABLE 5:** Rate of surgical care by race.

Cohort	Race	*N*	Rate of surgical care per 100 person‐years		Cox proportional hazard regression model	
			Rate (95% CI)	*p*	Unadjusted hazard ratio (95% CI)	Adjusted hazard ratio^a^ (95% CI)
**RP‐ED**	White	10 543	2.81 (2.61–3.02)	<0.0001*	Reference	Reference
	Black	1024	5.80 (4.91–6.85)		2.03 (1.69–2.44)	1.79 (1.49–2.17)
**RP‐UI**	White	11 168	7.10 (6.77–7.43)	0.0011*	Reference	Reference
	Black	932	5.67 (4.77–6.74)		0.84 (0.70–1.01)	0.80 (0.67–0.96)
**RT‐ED**	White	7225	1.41 (1.23–1.61)	0.0120*	Reference	Reference
	Black	1133	2.32 (1.80–2.99)		1.70 (1.28–2.26)	1.50 (1.11–2.01)
**RT‐UI**	White	4844	3.66 (3.28–4.09)	0.4567	Reference	Reference
	Black	485	3.15 (2.19–4.56)		0.88 (0.60–1.30)	0.72 (0.48–1.07)

Abbreviations: CI, confidence interval; ED, erectile dysfunction; IQR, interquartile range; RP, radical prostatectomy; RT, radiation therapy; UI, urinary incontinence; SD, standard deviation.

^a^ All statistically significant.

* Statistically significant at *p* ≤ 0.05.

**TABLE 6:** Rate of surgical care by income quartile.

Cohort	Income quartile	*N*	Rate of surgical care per 100 person‐years	
			Rate (95% CI)	*p*
**RP‐ED**	Q1	860	4.19 (3.38–5.21)	0.0153*
	Q2	1940	2.58 (2.16–3.09)	
	Q3	2814	3.08 (2.69–3.53)	
	Q4	5925	3.05 (2.78–3.35)	
**RP‐UI**	Q1	975	7.59 (6.52–8.85)	0.624
	Q2	2014	6.50 (5.80–7.29)	
	Q3	2974	7.10 (6.49–7.78)	
	Q4	6102	6.95 (6.53–7.41)	
**RT‐ED**	Q1	768	2.01 (1.45–2.08)	0.169
	Q2	1476	1.74 (1.34–2.25)	
	Q3	1892	1.50 (1.17–1.93)	
	Q4	4195	1.40 (1.18–1.66)	
**RT‐UI**	Q1	528	3.09 (2.17–4.41)	0.473
	Q2	877	4.13 (3.26–5.24)	
	Q3	1211	3.65 (2.92–4.56)	
	Q4	2704	3.52 (3.03–4.09)	

Abbreviations: CI, confidence interval; ED, erectile dysfunction; IQR, interquartile range; RP, radical prostatectomy; RT, radiation therapy; UI, urinary incontinence; SD, standard deviation.

* Statistically significant at *p* ≤ 0.05.

### 3.3 Cox proportional ‘time‐to‐surgical care’ regression analysis

**FIGURE 2:**
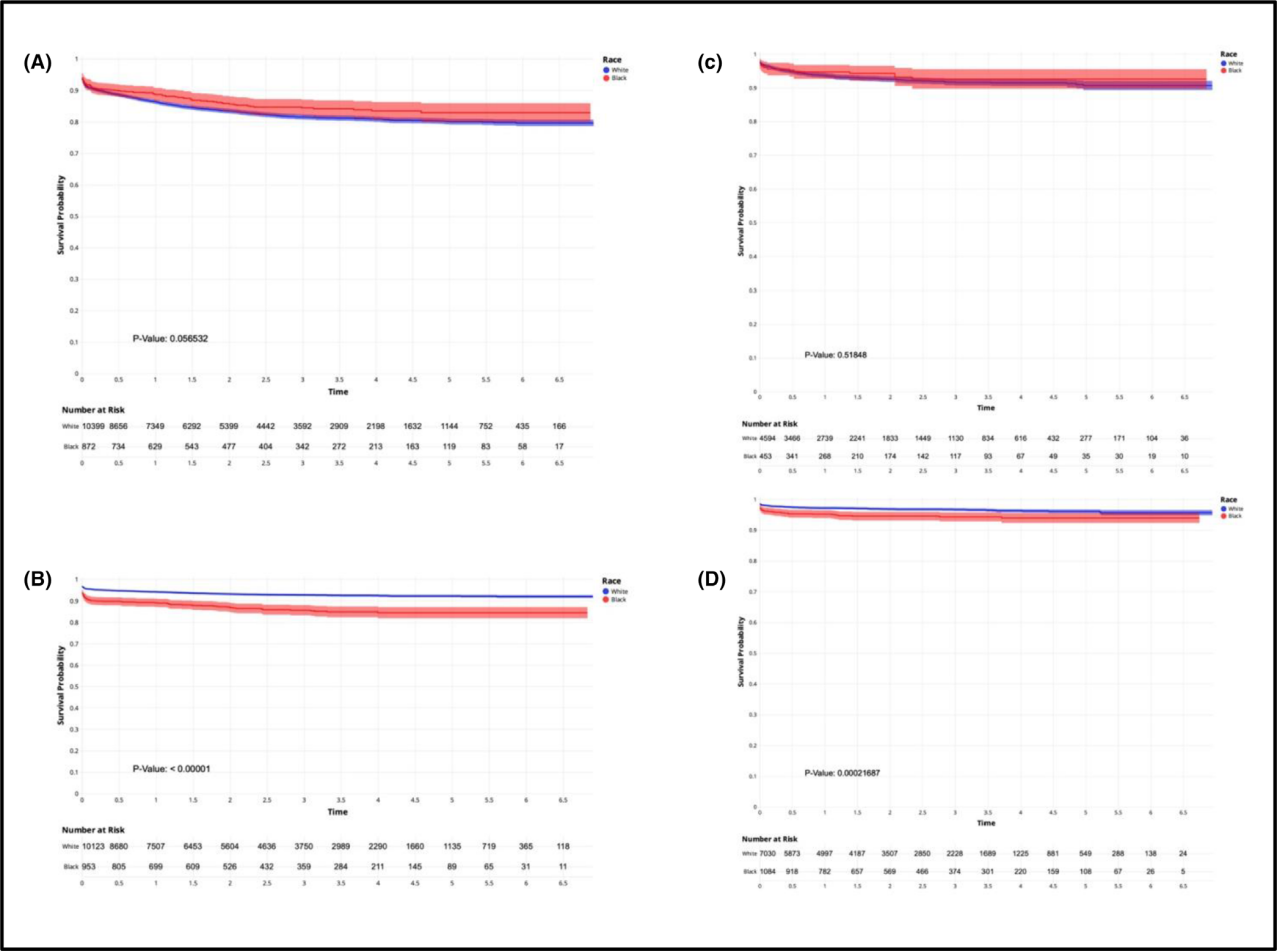
Kaplan–Meier (KM) survival plot of Cox proportional‐hazard regression model. (A) Radical Prostatectomy‐Urinary Incontinence (RP‐UI) cohort KM plot, (B) Radical Prostatectomy‐Erectile Dysfunction (RP‐ED) cohort KM plot, (C) Radiation Therapy‐Urinary Incontinence (RT‐UI) cohort KM plot and (D) Radiation Therapy‐Erectile Dysfunction (RT‐ED) cohort KM plot.

Cox proportional hazard regression analysis based on time from adverse events diagnosis to surgical care among men who received UI/ED treatment, reinforced the intra‐cohort comparison results, suggesting that Black men were more likely to receive ED surgical care (RP‐ED: Unadjusted Hazard Ratio [HR] 2.03, 95% CI:1.69–2.44; RT‐ED: HR 1.70, 95% CI: 1.28–2.26), but less likely to receive UI surgical care (RP‐UI: HR 0.84, 95% CI: 0.70–1.01; RT‐UI: HR 0.88, 95% CI 0.60–1.30), compared to White men. This pattern was statistically significant in all cohorts except RT‐UI, following covariate‐adjustment with age, patient census region and baseline Charlson co‐morbidity score (RP‐ED: Adjusted Hazard Ratio [AHR] 1.79, 95% CI: 1.49–2.17; RT‐ED: AHR 1.50, 95% CI: 1.11–2.01; RP‐UI: AHR 0.80, 95% CI: 0.67–0.96; Table 5 and Figure 2A–D).

## 4 DISCUSSION

This analysis revealed that overall rates of ED and UI surgical care among PCa survivors post‐treatment are low and differ by race and income status. In our study population (men ≥65 years), Black men were more likely to receive ED surgical care compared with White men. African American race has previously been shown to be a positive predictor of penile prosthesis implantation.^10^, ^11^ While a suitable explanation for this observation has been lacking in the literature, it may indicate a downstream consequence of delayed diagnosis and treatment, and the more advanced form of PCa seen in Black men,^12^ causing more severe ED patterns post‐treatment^13^ and requiring advanced care.^12^, ^13^

The rate of ED surgical care was found to be 3.1 and 1.5 per 100 person‐years, in the RP‐ED and RT‐ED cohorts respectively, equivalent to a prevalence rate of 7.4% (*n* = 852/11567) and 3.4% (*n* = 285/8358). Bajic et al.,^11^ using the Florida Healthcare Cost and utilization Project (HCUP) State Inpatient and Ambulatory database, reported that 4.9% (*n* = 1449/29288) of PCa patients who had RP subsequently received penile prosthesis implantation for ED. This is lower than estimates from our study but could be attributed to the different databases involved. While the HCUP database includes clinical data from uninsured patients, who may be unable to afford surgical care, this study used the SAF database, which excludes such patients, thus reducing the sample size significantly. Ultimately, the findings from this study, as well as existing literary evidence, suggest that ED surgical care is low, before consideration of racial, socioeconomic and demographic factors that influence receipt of care.

Overall, the rate of UI surgical care was 7.0 and 3.6 per 100 person‐years for the RP‐UI and RT‐UI cohorts, respectively. Recently, Nelson et al.^14^ estimated the prevalence of post‐RT UI surgical care among men within the Healthcare Cost and Utilization Project (HCUP) database to be 3.6% (*n* = 1068/29287). This is lower than the current study's RP‐UI cohort estimate of 15.7% (*n* = 1897/12100), but similar to the RT‐UI cohort estimate of 6.5% (*n* = 348/5329). These discrepancies again, may be attributed to the afore‐described limitations of the HCUP database's constitution.

Our results also indicate that White men were more likely to receive UI surgical care compared with Black men. Gupta et al.,^8^ reported similar findings, showing Black men received fewer UI corrective procedures compared to White men (2.1% vs 4.3%, *p* = 0.001). In another study, McAbee et al.^9^ reported that an overwhelmingly higher proportion of AUS and male sling insertions recorded within a single‐surgeon's database were performed on White men (9% vs. 87.2%, *p* = 0.018). While the racial disparities in receipt of ED and UI surgical care are puzzling, they remain substantial and have been observed outside the context of PCa diagnosis. In a cohort of men without PCa, Chen et al. found that African American men had a significantly higher probability of receiving ED treatment compared with Caucasian, Asian or Hispanic men.^15^ This may reflect cultural factors, that hypothetically make Black men sense a greater need to retain their sexual function, upon a diagnosis of ED following PCa treatment, compared with men from other racial groups.^16^, ^17^

The influence of income status on the receipt of surgical care was interesting, with men belonging to lower income quartiles having a higher rate of surgical care in most cohorts. This echoes the findings of Bajic et al.,^11^ who reported that men belonging to higher income quartiles were less likely to undergo penile prosthesis insertion (odds ratio [OR]: 0.8, *p* < 0.05) and more likely to have a longer time to penile prosthesis implantation (OR: 2.52, *p* < 0.01) than those belonging to lower income quartiles. This may be because other non‐invasive ED treatment options like alprostadil injections and vacuum erection devices are not routinely covered by Medicare,^18^, ^19^ narrowing the available Medicare‐covered treatments for men of lower income quartiles and making penile prosthesis implantation more favoured. However, the present study's use of geographical median household income figures as a proxy for income status may not accurately reflect individual income status for each patient.

ED and UI are the most common complications of PCa treatment and have been associated with significant decline in quality of life in over one‐third of patients following therapy.^20^, ^21^ These complications have been linked to depression, anxiety, frustration, and intimacy challenges,^22^ and early treatment is therefore critical to improving patient wellbeing and treatment satisfaction. The impact of racial and socioeconomic differences on surgical care for these complications exacerbate entrenched disparity trends, which are long‐established in PCa presentation patterns, diagnosis, treatment and outcomes.^23^ Interventions that improve uptake of ED and/or UI surgical care for men who need these procedures would contribute significantly to alleviating the disparity trends. This study employed rigorous methodological techniques such as Cox proportional‐hazard modelling to establish trends and statistical associations, while contributing fresh insights to a critical but sparsely investigated aspect of holistic PCa care, which could influence quality of care offered to PCa patients.

This study is, however, not without limitations, some of which are within the recognized limits of Medicare claims database utilization. By utilizing a Medicare database, this study excludes younger (<65 years) PCa patients, who are also significantly impacted by post‐treatment ED and/or UI. Additionally, claims that occurred prior to patients' eligibility and enrollment in fee‐for‐service Medicare were not available in the dataset, nor were any claims that occurred in settings outside the hospital. Given these limitations, initial ED and/or UI diagnosis occurring prior to fee‐for‐service Medicare enrollment or in a non‐hospital setting may not have been captured. Therefore, Cox regression analysis results were used to represent the directional association of time to definitive treatment for ED and/or UI. Also, we identified eligible PCa patients using relevant ICD‐9‐CM/ICD‐10‐CM codes, which do not differentiate between localized and metastatic PCa in the SAF database used. Thus, the presence of metastatic disease may represent additional confounders not accounted for in our analyses. We have limited our racial comparisons to Black and White patients as there was not a sufficient number of Hispanic and Asian patients captured within the SAF database and meeting our inclusion criteria to be included for statistical analysis, potentially excluding valuable insights specific to these racial groups. Finally, this study focused on surgical care and excluded other treatment options including PDE‐5i for ED and pelvic floor muscle training and penile compression clamps for UI because these options are not routinely covered within the Medicare database used. Inclusion of these treatment options may or may not alter the disparity trends presented.

## 5 CONCLUSION

Overall, uptake of surgical care for post‐PCa treatment complications is low and significantly impacted by racial and socioeconomic differences. Among men aged ≥65 years, Black men are more likely to receive ED surgical care, while White men are more likely to receive UI surgical care. Men of higher income quartiles had lower rates of ED or UI surgical care. Prospective and qualitative studies investigating the basis of these trends would be insightful.

## AUTHOR CONTRIBUTIONS

All of the authors met the following criteria:

- Made substantial contributions to the conception or design of the work; or the acquisition, analysis or interpretation of data for the work;
- Drafted the work or revised it critically for important intellectual content;
- Approved the final version to be published;
- Agreed to be accountable for all aspects of the work in ensuring that questions related to the accuracy or integrity of any part of the work are appropriately investigated and resolved.

## CONFLICTS OF INTEREST STATEMENT

Support for this research was provided by Boston Scientific, Marlborough, MA. Sirikan Rojanasarot and Alysha McGovern are full‐time employees of Boston Scientific. Oluwafolajimi Adesanya has no conflicts of interest to disclose. Arthur Burnett is a research award recipient from Boston Scientific. Oluwafolajimi Adesanya and Arthur Burnett were not compensated for their participation in this study.

## Data Availability

Due to data use agreements signed with CMS, the data cannot be provided externally. Other researchers can purchase the same dataset to carry out similar analyses.
